# Hierarchical Ordering Induced Ultrahigh Cryogenic Strength and Strain Hardening in a Ni_2_CoFeV Medium‐Entropy Alloy

**DOI:** 10.1002/advs.75679

**Published:** 2026-05-11

**Authors:** Lei Gu, Wei Jiang, Qingzhong Mao, Xiang Chen, Yonghao Zhao

**Affiliations:** ^1^ School of Materials Science and Engineering Hohai University Changzhou China; ^2^ Jiangsu Provincial Engineering Research Center for Structure‐Function Integrated Metallic Materials For Harsh Environments School of Materials Science and Engineering Hohai University Changzhou China; ^3^ Innovation Center For Critical Materials in Hydraulic Infrastructure Safety and Water Environment Restoration School of Materials Science and Engineering Hohai University Changzhou China; ^4^ School of Materials Science and Engineering Anhui Polytechnic University Wuhu China; ^5^ Nano and Heterogeneous Materials Center School of Materials Science and Engineering Nanjing University of Science and Technology Nanjing China; ^6^ Institute of Materials Henan Academy of Sciences Zhengzhou China

**Keywords:** κ phase, cryogenic temperatures, L1_2_ phase, medium‐entropy alloys, strain hardening, strength and ductility

## Abstract

Conventional metallic materials for cryogenic engineering are typically single‐phase face‐centered cubic (fcc) alloys with limited yield strength, whereas ordered phases enhance strength at the expense of ductility, in some cases even causing a ductile‐to‐brittle transition. Here, we overcome this long‐standing limitation by designing a non‐equiatomic fcc Ni_2_CoFeV medium‐entropy alloy featuring hierarchically ordered, intragranular κ and L1_2_ intermetallic phases. The resulting tri‐phase alloy achieves a high yield strength of 1.4 GPa at 77 K, enabled by severe lattice distortion from V enrichment together with synergistic strengthening from coherent phase interfaces. Notably, exceptional strain hardening, characterized by an ultrahigh strain hardening rate of ∼7 GPa and a large exponent of 0.85, drives a tensile strength exceeding 2 GPa while retaining a high ductility of 28% at 77 K, surpassing that of most reported fcc‐based high‐/medium‐entropy alloys. The superior cryogenic performance arises from the sequential activation of multiple deformation mechanisms, involving high‐density superlattice dislocations and stacking faults in the coupled fcc/L1_2_ phases and dislocation multiple slip in the κ phase. This work establishes hierarchical intermetallic ordering as an effective paradigm for cryogenic materials design.

## Graphical abstract

A tri‐phase Ni_2_CoFeV medium‐entropy alloy with fcc, κ, and L1_2_ phases achieves exceptional cryogenic strength‐ductility synergy at 77 K. It delivers a yield strength of 1.4 GPa, an ultimate tensile strength of 2.07 GPa and a tensile ductility of 28%. Activated multiple deformation mechanisms enable superior strain hardening, establishing a robust strategy for designing ultrastrong cryogenic metallic materials.

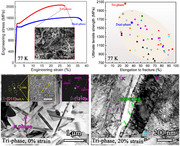

## 1 Introduction

Metallic materials with high strength and good ductility at cryogenic temperatures are essential for advanced engineering applications, such as liquefied natural gas storage and transportation, the Chang'e lunar probe, and nuclear fusion reactor. Single‐phase face‐centered cubic (fcc) metals and alloys are traditionally favored for cryogenic applications because of their intrinsic resistance to the ductile‐to‐brittle transition. Representative examples include 300‐series austenitic stainless steels and high‐Mn steels, which exhibit high ultimate tensile strength (UTS) and excellent ductility at cryogenic temperatures [1, 2, 3, 4]. Their superior strain hardening capability originates from additional deformation mechanisms such as deformation twinning and phase transformation, associated with twinning‐induced plasticity (TWIP) and transformation‐induced plasticity (TRIP) effects. Despite these advantages, the yield strength (YS) of traditional cryogenic metallic materials remains relatively low. In contrast, many conventional high‐strength materials that perform well at room temperature suffer from pronounced brittleness when exposed to cryogenic conditions [5, 6].

Over the past two decades, multi‐principal element alloys, known as high‐/medium‐entropy alloys (HEAs/MEAs), have emerged as promising candidates for overcoming this limitation [7, 8, 9, 10]. Among them, the prototypical equiatomic CoCrFeMnNi HEA and its derivative CoCrNi MEA, exhibit exceptional fracture toughness at 77 K and even 20 K, outperforming most traditional metallic materials [11, 12, 13]. Furthermore, both alloys possess high UTS values of ∼1.3 GPa and fracture elongations exceeding 77% at 77 K [11, 12], enabled by a pronounced TWIP effect that promotes the formation of a stable 3D twin network during deformation [14]. Unfortunately, their YS remains below 800 MPa, limiting their applicability in extreme cryogenic load‐bearing environments. For certain metastable fcc HEAs/MEAs, cryogenic temperatures can enhance the TRIP effect, triggering a phase transformation from fcc matrix to bcc (body‐centered cubic) or hcp (hexagonal close‐packed) phases [15, 16, 17]. While such transformations provide substantial dynamic strengthening, they contribute little to improving the initial YS.

Conventional strengthening strategies, including solid‐solution, grain boundary (GB), dislocation, and precipitation strengthening, have therefore been employed to improve the YS of HEAs/MEAs. For example, the addition of V element with a larger atomic size induces severe lattice distortion in single‐phase fcc CoNiV MEAs, which gives rise to high friction stress and contributes to a room‐temperature YS approaching 1 GPa [18]. This effect is further amplified at cryogenic temperatures due to the increased Peierls barrier [19]. However, GB and dislocation strengthening often compromise ductility and strain hardening capability by restricting dislocation mobility and storage [20, 21, 22]. Similarly, the introduction of precipitates, such as the L1_2_, bcc and B2 phases can further increase YS, yet precipitation‐strengthened HEAs/MEAs frequently suffer from cryogenic brittleness [23, 24].

In our previous work, we developed a Ni_2_CoFeV (at%) MEA with an outstanding combination of ultrahigh strength and ductility at room temperature by introducing hierarchically ordered phases [25]. Specifically, coherent κ‐type (Co, Fe, Ni)_3_V and L1_2_‐type (Co, Fe, Ni)_3_(V, Fe) intermetallic phases were embedded within a disordered fcc matrix [25]. Building on this concept, the present study investigates whether such low‐symmetry ordered phases can enhance strength at cryogenic temperatures without introducing a ductile‐to‐brittle transition. The tri‐phase MEA achieves a high YS of 1.4 GPa, while the sequential activation of multiple deformation mechanisms sustains exceptional strain hardening, leading to an ultrahigh UTS exceeding 2 GPa and a fracture elongation of 28%.

## 2 Results

### 2.1 Initial Microstructures

**FIGURE 1:**
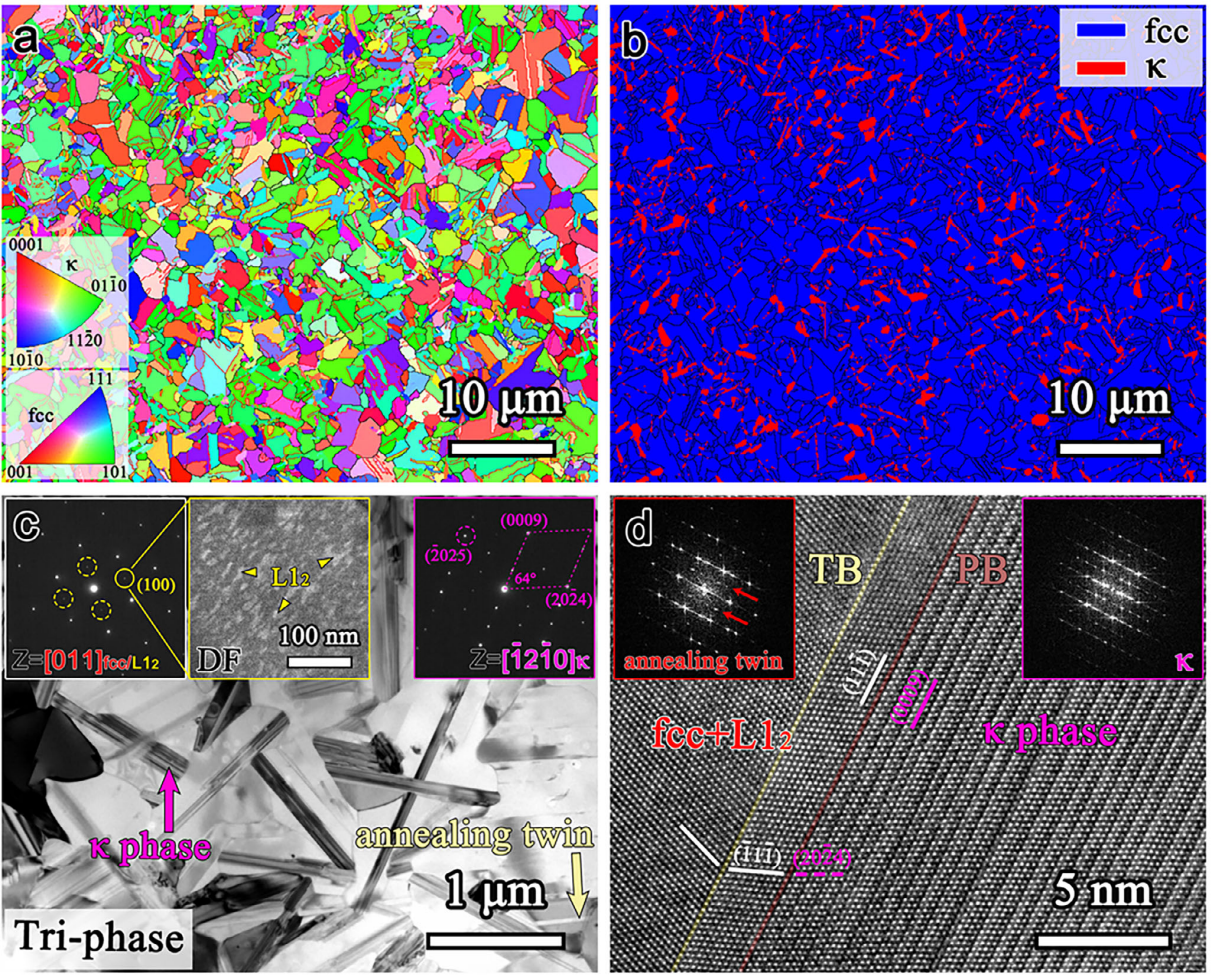
Microstructures of the tri‐phase Ni_2_CoFeV MEA. (a, b) Electron backscattered diffraction (EBSD) crystal orientation and phase maps. The high‐angle GBs (HAGBs: > 15°) are marked by black lines in (a) and (b). The twin boundaries (TBs: 60°) are marked by red lines in (a). (c) TEM image with the inserted SAED patterns of the coupled fcc/L1_2_ phases and the κ phase. The inserted dark‐field (DF) image recorded from (100) superlattice spot in (c) shows the morphology of the L1_2_ phase. (d) High‐resolution TEM (HRTEM) image and fast Fourier transform (FFT) patterns showing the atomic and crystal structures of the κ and fcc/L1_2_ phases. PB: phase boundary.

Microstructural characterization reveals that recrystallization annealing at 750°C for 10 min combined with aging at 650°C for 3 h has produced a tri‐phase structure in the Ni_2_CoFeV MEA, as shown in Figure 1. The fully recrystallized fcc grains are near‐equiaxed and exhibit random crystal orientations (Figure 1a,b), with a size ranging from submicron to several microns and an average grain size of 1.22 µm (Figure S1a). Within the fcc grains, plentiful annealing twins (marked by red lines in Figure 1a) and ultrafine lath‐shaped κ phase (Figure 1b) are detected traversing the entire fcc grains. The average equivalent circular diameter of the κ phase is 527 nm (Figure S1b). In transmission electron microscopy (TEM) images, the κ phase and annealing twin are readily distinguishable, owing to the prominent light‐dark contrast within the κ phase (Figure 1c). Additionally, both the fcc and κ phases possess an extremely low dislocation density. The κ phase exhibits a volume fraction of ∼13%, an average length of 1.08 µm, and a width of 121 nm (Figure S1c,d). Based on the selected‐area electron diffraction (SAED) patterns in Figure S2a, the κ phase exhibits an orientation relationship of (0009)κ//(11$\bar{1}$)fcc and [$\bar{1}$2$\bar{1}$0]κ//[011]fcc with the fcc matrix. Meanwhile, the precipitation of L1_2_ phase occurs within the fcc matrix, confirmed by the faint superlattice diffraction spots under the [011] zone axis (Figure 1c), compared to the fcc phase (Figure S3b). The inserted dark‐field image recorded from (100) superlattice spot indicates the irregular L1_2_ nanoprecipitates are dispersed in the fcc matrix, with a volume fraction of ∼24%. Thus, a tri‐phase structure, namely fcc + κ + L1_2_ phases, is generated in the Ni_2_CoFeV MEA. Notably, the fcc grains and κ phase in the tri‐phase MEA barely coarsen, and the volume fraction of the κ phase remains nearly unchanged, compared to the dual‐phase MEA (Figure S3). Figure 1d further illustrates a perfect coherent interface between the κ and fcc/L1_2_ phases, with an orientation relation of (0009)κ//(11$\bar{1}$)fcc/L1_2_ and [$\bar{1}$2$\bar{1}$0]κ//[011]fcc/L1_2_ (Figure S2b). Another ($\bar{1}$1$\bar{1}$) plane of the fcc/L1_2_ phases has an angle relationship of ∼6.5° with the (20$\bar{2}$4) plane of the κ phase. Moreover, the κ phase exhibits high‐density planar defects lying in a plane perpendicular to the [$\bar{1}$2$\bar{1}$0] viewing direction (Figure 1d), deviating from perfect atomic stacking with a nine‐layered abcbcacab sequence [26, 27]. Local lattice distortions in both the fcc/L1_2_ matrix and κ phase were quantitatively characterized via displacement separation analysis (DSA), as presented in Figure S4. The DSA results demonstrate that the fcc/L1_2_ matrix possesses low‑amplitude, randomly distributed lattice distortions. In contrast, the κ phase exhibits substantially larger, anisotropically correlated lattice displacements, indicating a higher internal strain field within the κ phase. The X‐ray diffraction (XRD) patterns further demonstrate that the κ phase exists in both dual‐phase and tri‐phase MEAs (Figure S5). However, there is no additional diffraction peak corresponding to the L1_2_ phase in the tri‐phase MEA, due to its close similarity to the disordered fcc phase. Notably, precipitation of the L1_2_ phase leads to a reduction in the lattice constant, resulting in a shift of the fcc diffraction peaks to higher angles.

### 2.2 Cryogenic Mechanical Properties

**FIGURE 2:**
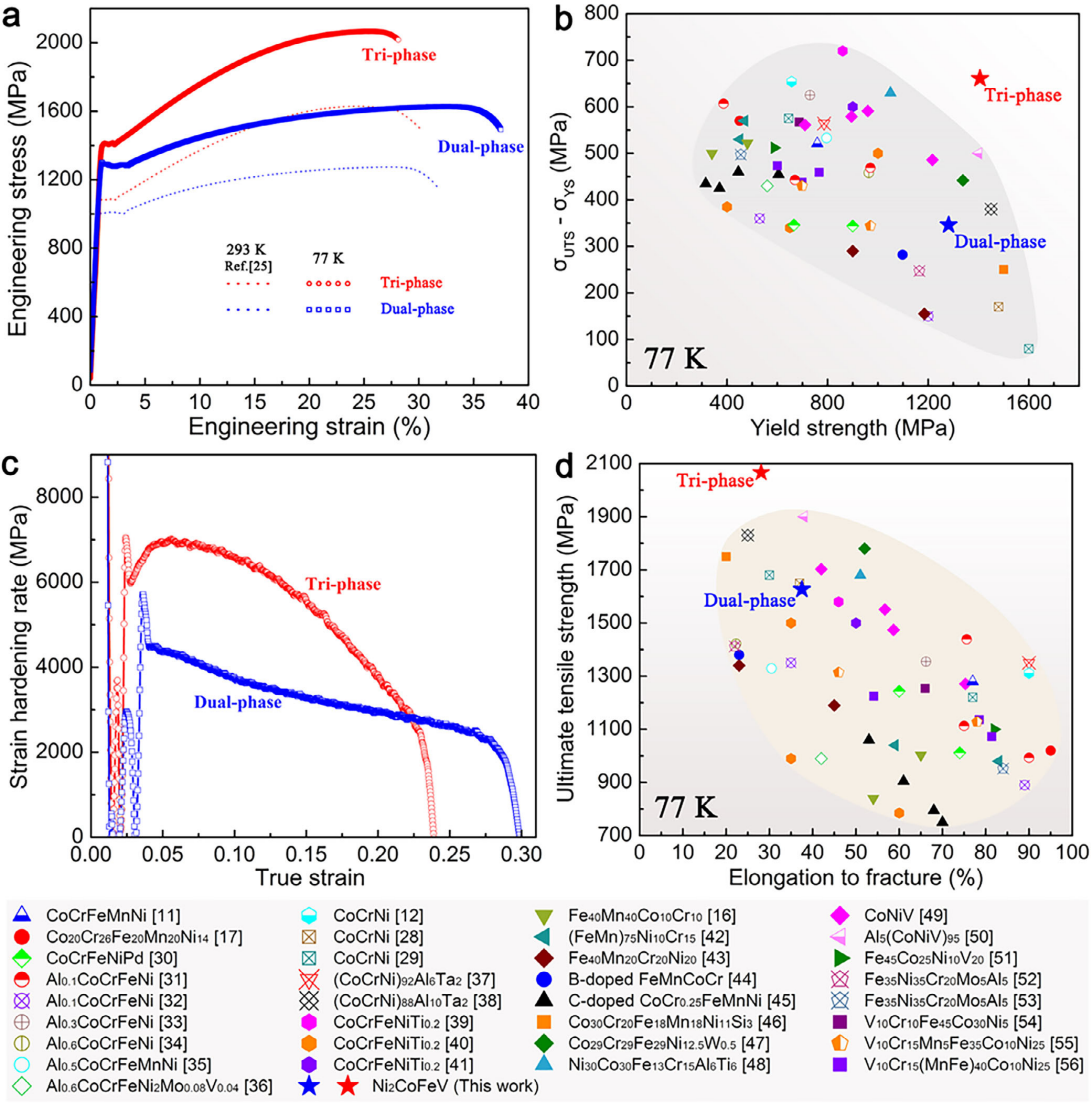
Cryogenic mechanical properties of the dual‐phase and tri‐phase Ni_2_CoFeV MEAs at 77 K. (a) Tensile engineering stress–strain curves. The dotted lines in (a) showcase the curves at 293 K for reference [25]. (b) Comparison of strain hardening capacity (*σ*_UTS_ ‐ *σ*_YS_) versus YS of our MEAs (blue and red stars) with most fcc‐based HEAs/MEAs reported previously [11, 12, 16, 17, 28, 29, 30, 31, 32, 33, 34, 35, 36, 37, 38, 39, 40, 41, 42, 43, 44, 45, 46, 47, 48, 49, 50, 51, 52, 53, 54, 55, 56]. (c) The corresponding SHR and true strain relations. (d) Comparison of UTS versus elongation [11, 12, 16, 17, 28, 29, 30, 31, 32, 33, 34, 35, 36, 37, 38, 39, 40, 41, 42, 43, 44, 45, 46, 47, 48, 49, 50, 51, 52, 53, 54, 55, 56].

Figure 2a displays the representative tensile engineering stress–strain curves of the dual‐phase and tri‐phase Ni_2_CoFeV MEAs tested at 77 K. For comparison, the corresponding curves of these two Ni_2_CoFeV MEAs tested at room temperature (293 K) are included [25]. Both the MEAs exhibit a high YS exceeding 1.2 GPa at 77 K, representing an increase of ∼300 MPa relative to their respective YS at 293 K. Accurately, the YSs are 1.28 GPa for the dual‐phase MEA and 1.4 GPa for the tri‐phase MEA, respectively. After yielding, the stress of the dual‐phase MEA increases with strain, attaining a UTS of ∼1.63 GPa at a total elongation of 37.5%. In contrast, the tri‐phase MEA achieves a drastically enhanced UTS of ∼2.07 GPa alongside a ductility of 28.1%. Ductile fracture behavior is clearly evidenced by the uniformly distributed, honeycomb‐like dimples observed on the fracture surfaces (Figure S6). The difference between the UTS and YS of the tri‐phase MEA is 660 MPa (Figure 2b), demonstrating its extraordinary strain hardening capacity at such a high YS level. As shown in Figure 2c, the tri‐phase MEA exhibits an ultrahigh strain hardening rate (SHR, *Θ* = ∂*σ*/∂*ε*, where *σ* and *ε* denote true stress and true strain, respectively), featuring a transient up‐turn subsequent to the initial elasto‐plastic transition region. A peak SHR of 7.01 GPa is achieved, with values remaining above 5 GPa up to a true strain of ∼0.16. In comparison, the SHR of the dual‐phase MEA consistently remains below 5 GPa throughout plastic deformation. The strain hardening exponent (*n*), derived from the Ludwick equation ($\sigma_{T}=\sigma_{0}+K\varepsilon_{T}^{n}$, where σ_T_, σ_0_, *K*, and ε_T_ represent true stress, YS, strengthening coefficient, and true plastic strain, respectively), can also be used to evaluate the extent of strain hardening. By fitting the full‐range true stress‐true plastic strain curves, the overall *n* values of the tri‐phase and dual‐phase MEAs are deduced to be 0.85 and 0.82, respectively (Figure S7a). Segmented fitting analysis shows that the *n* value of the dual‐phase MEA slightly decreases from 0.89 in the initial stage (ε_T_: 0–0.1) to 0.79 in the later stage (ε_T_: 0.15–0.2) (Figure S7b–d). While the tri‐phase MEA shows a higher initial *n* value of 1.01, followed by a pronounced drop to 0.64 in the later stage. Notably, the tri‐phase MEA exhibits not only high YS but also exceptional strain hardening capacity, which effectively offsets the loss of load‐bearing capacity induced by geometric softening. This superior characteristic enables the tri‐phase MEA to achieve both ultrahigh UTS and excellent ductility at 77 K, surpassing most previously reported fcc‐based HEAs/MEAs in terms of strength (Figure 2d) [11, 12, 16, 17, 28, 29, 30, 31, 32, 33, 34, 35, 36, 37, 38, 39, 40, 41, 42, 43, 44, 45, 46, 47, 48, 49, 50, 51, 52, 53, 54, 55, 56]. These findings validate the effectiveness of the hierarchical ordered‐phase strengthening strategy in simultaneously improving strength and preserving ductility at cryogenic temperatures.

### 2.3 Cryogenic Deformation Mechanisms

**FIGURE 3:**
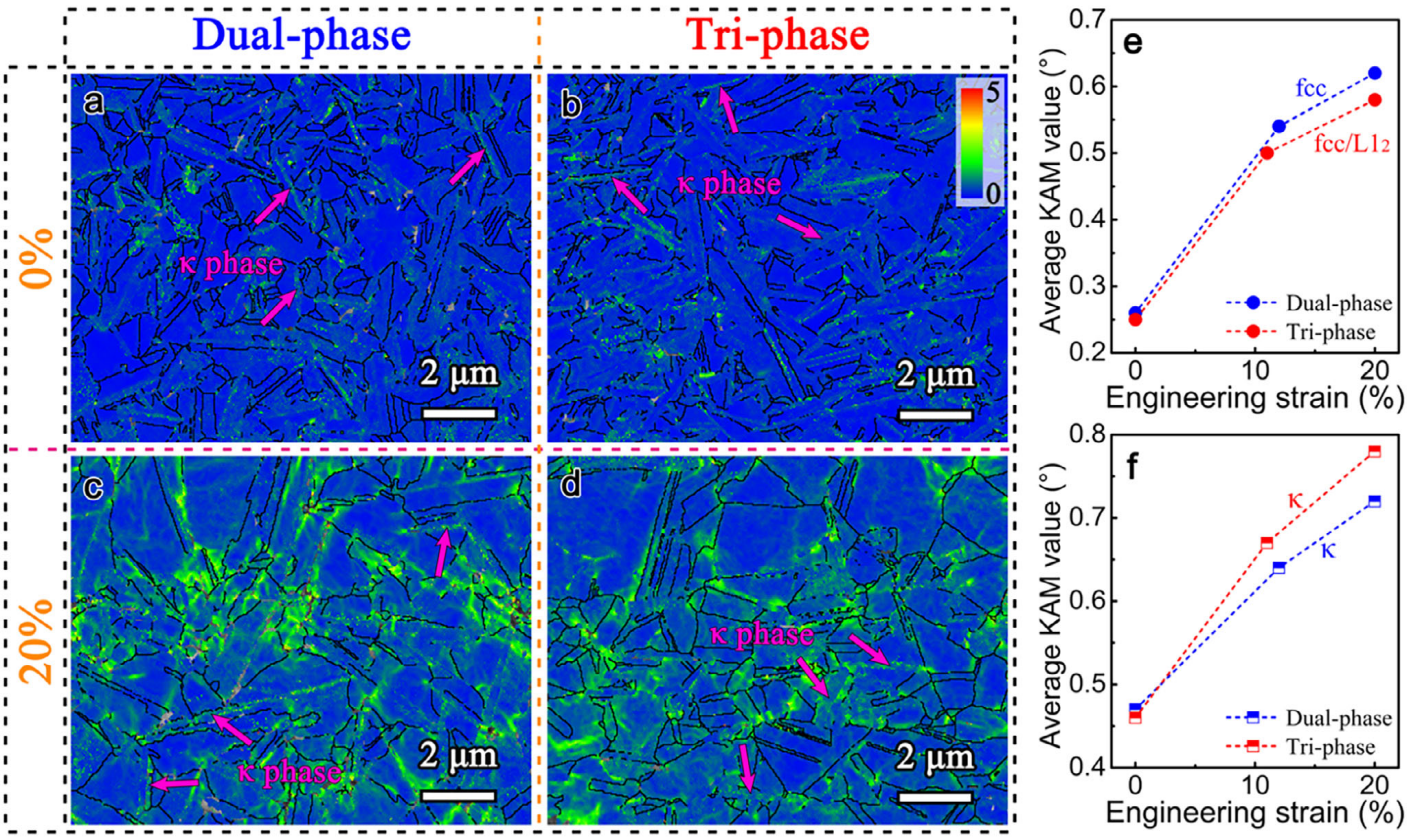
Transmission Kikuchi diffraction (TKD) local misorientation maps of the dual‐phase and tri‐phase MEAs tested at 77 K with different engineering strains. (a, b) 0%, and (c,d) 20%. The HAGBs of fcc grains are marked by black lines in (a–d). (e, f) The evolution of the average KAM values for the fcc/L1_2_ and κ phases.

Figure 3a–d and Figure S8 present local misorientation maps for characterizing the evolution of geometrically necessary dislocation (GND) density during tensile deformation, as the latter exhibits a linear relationship with the Kernel average misorientation (KAM) [57, 58, 59]. The color bar in Figure 3b, ranging from blue to red, indicates an increasing trend of KAM values, with a threshold value of 5°. The evolution of KAM values with strain and their distributions at different strains are presented in Figure 3e,f and Figure S9, respectively. Both the dual‐phase and tri‐phase MEAs exhibit a gradual increase in GND density with increasing tensile strain. Prior to tensile deformation, the κ phase and matrix in the dual‐phase MEA exhibit nearly identical dislocation densities to those in the tri‐phase MEA. Under identical strain conditions, the GND density of the fcc matrix in the dual‐phase MEA is higher than that of fcc/L1_2_ matrix in the tri‐phase MEA, while the opposite is true for the κ phase. Moreover, the harder κ phase exhibits a much slower GND density growth rate than the fcc and fcc/L1_2_ matrix, regardless of in the dual‐phase or tri‐phase MEAs. Specifically, when tensile strain increases from 0% to 20%, the GND density increases by 138% for the fcc matrix and 53% for the κ phase in the dual‐phase MEA, and by 132% for the fcc/L1_2_ matrix and 70% for the κ phase in the tri‐phase MEA, respectively. While at necking, the dual‐phase MEA exhibits a higher GND density than the tri‐phase MEA, mainly because it undergoes a higher tensile strain (Figure S8c,d).

**FIGURE 4:**
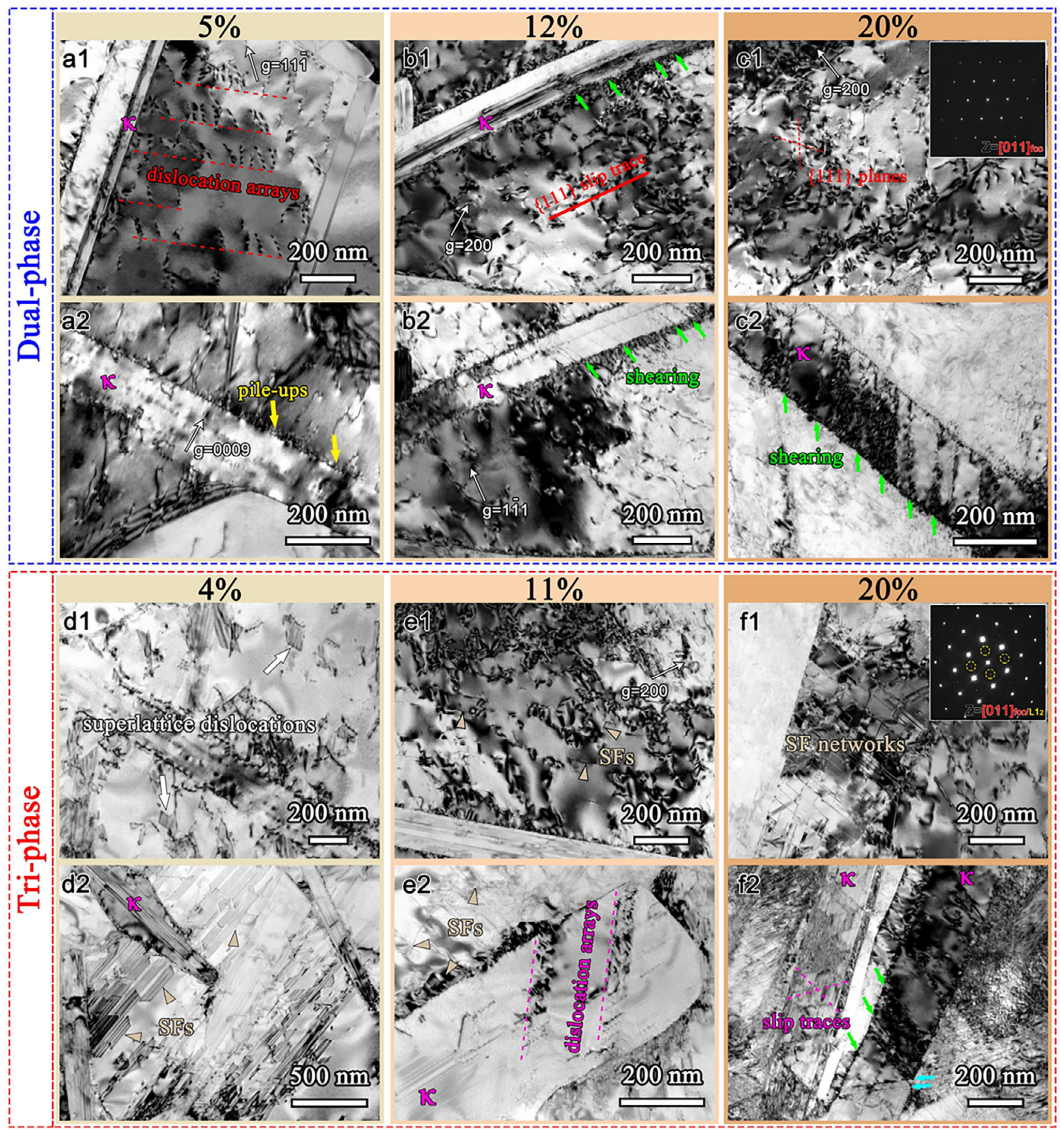
TEM images and corresponding SAED patterns, showing the deformation microstructures deformed at 77 K with different engineering strains. (a1–c2) The dual‐phase MEA at strains of (a1, a2) 5%, (b1, b2) 12%, and (c1, c2) 20%. (d1–f2) The tri‐phase MEA at strains of (d1, d2) 4%, (e1, e2) 11%, and (f1, f2) 20%. The paired superlattice dislocations are marked by white arrows in (d1). The SFs in the coupled fcc/L1_2_ phases are marked by triangles.

To further investigate the underlying deformation mechanisms, post‐mortem TEM observations were conducted on the dual‐phase and tri‐phase MEAs deformed to different tensile engineering strains at 77 K. Figure 4a1–c2 shows the deformation microstructures of the dual‐phase MEA at 5%, 12%, and 20%. At an initial strain of 5%, planar dislocation slip is observed, characterized by numerous parallel dislocation arrays in the fcc phase, and some piling up at twin boundaries (Figure 4a1). Dislocations are activated in the fcc phase and accumulate at the fcc/κ phase boundaries (marked by yellow arrows in Figure 4a2), indicating that the κ phase exerts strong resistance to dislocation slip. At a strain of 12%, the dislocation density increases obviously, leading to dislocation interactions and the formation of {111} slip traces in the fcc phase (Figure 4b1,2). Moreover, the κ phase also undergoes plastic deformation, with notable dislocation shearing (marked by green arrows in Figure 4b1,2). With further strain increase to 20%, two sets of {111} slip traces in the fcc phase confirm the activation of multiple slip systems (Figure 4c1). Additionally, dislocation shearing along the single slip system of the κ phase generates numerous parallel dislocation arrays with nanoscale interval space (Figure 4c2). After a tensile fracture, high dense dislocations in the fcc phase undergo mutual reactions and entanglement, while dislocation shearing in the κ phase induces the formation of sub‐boundaries (Figure S10).

Figure 4d1–f2 presents the deformation microstructures of the tri‐phase MEA at engineering strains of 4%, 11%, and 20%. As shown in Figure 4d1,2, plentiful paired superlattice dislocations and parallel SFs are observed in the coupled fcc/L1_2_ phases under 4% strain. At 11% strain, a higher density of dislocations and SFs is generated (Figure 4e1). The blocking of dislocation motion by the κ phase leads to the accumulation of high‐density dislocations at the phase boundaries, wherein some dislocations penetrate the κ phase (Figure 4e2). At 20% strain, abundant nano‐SF networks, with an average spacing of less than 50 nm, and dislocation tangles are formed in the coupled fcc/L1_2_ phases (Figure 4f1). Notably, multiple slip systems characterized by two sets of dislocation slip traces are activated in the κ phase (Figure 4f2). After a tensile fracture, dense SF networks and accumulated dislocations are observed in the coupled fcc/L1_2_ phases (Figure S11). However, no deformation twins are detected. Dislocation shearing on the dual slip systems induced segmentation of the κ phase.

## 3 Discussion

As described above, via a hierarchical ordered‐phase architecture, we have achieved an unprecedented combination of high YS, exceptional strain hardening, ultrahigh UTS, and large ductility in the tri‐phase Ni_2_CoFeV MEA at 77 K.

### 3.1 High YS at 77 K

First, we discuss the fundamental mechanisms governing the high YS of the tri‐phase Ni_2_CoFeV MEA. Generally, the high YS is associated with contributions from friction stress σ_0_, dislocation strengthening σ_dis_, GB strengthening σ_gb_ and phase boundary strengthening σ_pb_. Notably, in a single‐phase fcc CoNiV MEA, the synergistic effects of high friction stress and GB strengthening contribute to a YS approaching 1 GPa [18]. The high friction stress is nearly threefold greater than that of the reported MEAs/HEAs and traditional austenitic steels, a phenomenon attributed to severe lattice distortion caused by the solid solution of V atoms [18]. Annealing treatment induces the formation of the κ phase in the CoNiV MEA. Compared with the fcc matrix, the κ phase possesses a higher average nanohardness and modulus, and acts as the strongest contributor to the outstanding mechanical properties, with a YS of 1100 MPa and a UTS of 1550 MPa [60]. In our previous work, the tri‐phase Ni_2_CoFeV MEA, with a reduced content of the noble metal V, also achieves a gigapascal‐level YS at 293 K through the manipulation of a hierarchical ordered‐phase microstructure [25]. The gigapascal YS stems from the synergistic effects of high friction stress (arising from the solute concentration of V) and strengthening contributions from a high density of GBs and κ phase.

As the temperature decreases from 293 and 77 K, the YS of the tri‐phase MEA increases by over 300 MPa to 1.4 GPa (Figure 2a). Few dislocations are observed in the tri‐phase Ni_2_CoFeV MEA (Figures 1c and 3b), thus Δσ_dis_ can be disregarded. Moreover, Δσ_gb_ and Δσ_pb_ are classified as athermal components, neither of which contributes to the YS increment within the temperature range of 77–293 K [61, 62, 63]. By contrast, the friction stress Δσ_0_ is a thermal component sensitive to deformation temperature and serves as the primary contributor to the YS increment of the tri‐phase MEA at 77 K.

Notably, the tri‐phase Ni_2_CoFeV MEA exhibits a higher YS than the dual‐phase MEA at 77 K. Neither the fcc grains nor the κ phase in the tri‐phase MEA undergoes significant coarsening, and the volume fraction of the κ phase remains nearly unchanged (Figures S1 and S3). Thus, the enhancement of YS mainly stems from the precipitation of the L1_2_ phase in the fcc matrix (Figure 1c). It should be clear that the precipitation of the L1_2_ phase inevitably alleviates the lattice distortion of the FCC matrix, while increasing the density of the fcc/L1_2_ phase boundaries.

### 3.2 Exceptional Strain Hardening, Ultrahigh UTS and High Ductility at 77 K

We now elaborate on the underlying factors underpinning the exceptional strain hardening of the tri‐phase MEA, which in turn facilitates its ultrahigh UTS and excellent ductility at 77 K. First, the fcc/κ phase boundaries and the additional fcc/L1_2_ phase boundaries act as effective barriers to dislocation slip, thereby conferring enhanced strain hardening in the tri‐phase MEA. Second, the high V atom concentration induces a strong solute drag effect, significantly improving the dislocation storage capacity of the tri‐phase MEA. For the dual‐phase MEA, slip band composed of dislocation arrays in the fcc phase (Figure 4a1) are indicative of short‐range ordering (SRO) structures [64, 65]. These SRO structures impose local resistance on the movement of the leading dislocation; however, they simultaneously create less‐resistance channels for the subsequent dislocation movement. In contrast, the tri‐phase MEA is featured by paired superlattice dislocation structures (Figure 4d1). This is because the movement of paired superlattice dislocation (Figure 4d1) does not disrupt the long‐range ordered structure of the L1_2_ phase, thus retaining their ability to hinder the dislocations [66].

Third, deformation‐induced SFs generate in the coupled fcc/L1_2_ phases (Figure 4d2). The interaction between glide dislocations and SFs hinders the motion of dislocations on slip planes intersecting the SF planes, serving as a key contributor to strain hardening [67]. At the late stage of plastic deformation (20% strain), intersecting SFs on different {111} slip systems form nanoscale SF networks and immobile Lomer–Cottrell locks, which in turn reduce the dislocation mean free path [31, 48]. Stair‐rod dipoles within Lomer‐Cottrell locks exhibit a strong attractive force, inducing a high stress field that further impedes dislocation motion [68, 69]. Furthermore, Lomer‐Cottrell lock networks can act as Frank‐Read sources to promote dislocation multiplication [70]. Abnormally, despite the high density of SFs observed after tensile fracture at 77 K (Figure S11), no deformation twins are detected, even though the stacking fault energy (SFE) is lower than that at room temperature [25]. This implies that the critical twinning stress of the coupled fcc/L1_2_ phases is sufficiently high and remains unreached under the present tensile loading, even when the true stress exceeds 2.6 GPa (Figure S7a). A similar twinning inhibition phenomenon has also been observed in the ordered‐phase strengthened FeCoNiCrTi_0.2_ and Ni_30_Co_30_Fe_13_Cr_15_Al_6_Ti_6_ HEAs with lower SFE [39, 48]. Notably, the critical twinning stress in the L1_2_‐strengthened FeCoNiCrTi_0.2_ HEA was predicted to be approximately 6600 MPa at 77 K [39]. The suppression of twinning can be rationalized by the presence of the coherent L1_2_ ordered phase. Specifically, the glide of partial dislocations on successive {111} planes becomes energetically unfavorable, as such motion would disrupt the ordered L1_2_ structure and generate a high density of high‐energy anti‐phase boundaries. Consequently, deformation twinning becomes more difficult in the tri‐phase MEA compared to conventional low‐SFE alloys without L1_2_ nanoprecipitates.

The κ phase in the tri‐phase MEA frequently undergoes multiple dislocation slip on different planes and exhibits a higher dislocation density, resulting in additional dislocation forest hardening. Compared to the dual‐phase MEA, the significantly enhanced matrix strength in the tri‐phase MEA induced by the fcc/L1_2_ phase interfaces and dynamically generated SFs improves the dislocations shearing capability of the κ phase. The perfect coherent interfaces between the κ and fcc/L1_2_ phases facilitate the optimal distribution of strain and stress, and continuous dislocation shearing of the κ phase effectively relieves stress concentration at phase boundaries, both decreasing the likelihood of damage nucleation along the interface. In summary, multiple strain hardening mechanisms are activated successively in the tri‐phase MEA, providing the sustainable high SHR. Eventually, premature necking instability caused by strain localization is suppressed, enabling an unparalleled combination of ultrahigh UTS and excellent ductility at liquid nitrogen temperature.

## 4 Conclusions

A tri‐phase Ni_2_CoFeV MEA with hierarchical ordered‐phases, including the fcc, L1_2,_ and κ phases, is developed for cryogenic load‐bearing applications. This alloy exhibits ultrahigh cryogenic strengths, with a YS of 1.4 GPa and a UTS of 2.07 GPa, along with excellent tensile elongation of 28% at 77 K. Notably, contrary to conventional high‐YS alloys, the tri‐phase MEA demonstrates an extraordinary strain hardening capacity, superior to most reported fcc single‐phase and fcc‐based multi‐phase HEAs/MEAs. Systematic TEM observations at different tensile strains uncover that the strong interaction of dislocations and SFs in the coupled fcc/L1_2_ phases substantially elevates the matrix strength, consequently triggering dislocation shearing on multiple slip planes in the κ phase. These unexpected multiple deformation mechanisms contribute to the ultrahigh strain hardening, thereby achieving a combination of ultrahigh UTS and excellent ductility. This work establishes hierarchical multicomponent intermetallic architectures as a powerful strategy for designing ultrastrong yet ductile HEAs/MEAs for cryogenic applications.

## 5 Experimental Section

### 5.1 Sample Preparation

The as‐cast Ni_2_CoFeV MEA was fabricated by vacuum induction melting. Multi‐directional forging at room temperature, followed by annealing at 1150°C for 6 h, refined the millimeter‐sized grains. The bulk was then cut into plates and cold‐rolled with a ∼90% thickness reduction. A dual‐phase MEA was prepared by recrystallization annealing at 750°C for 10 min. Further aging at 650°C for 3 h formed the tri‐phase MEA. All heat‐treated samples were rapidly quenched in water.

### 5.2 Microstructural Characterization

EBSD and TKD measurements were performed using a TESCAN CLARA scanning electron microscopy (SEM). The EBSD and TKD data were analyzed with commercial channel 5 software, considering boundaries with misorientation angles >15° as HAGBs. TKD scanning step size was chosen as 30 nm for all samples. The filter size of 5 × 5 was applied to a smooth local misorientation map. TEM and high‐resolution TEM were conducted on a FEI‐Tecnai G2 20 S‐TWIN microscope at 200 kV and a Titan G2 60–300 microscope at 300 kV, respectively. Quantitative analysis of local lattice distortion in HRTEM images was performed using DSA [71]. Atomic lattice sites were first localized by 2D Gaussian fitting of periodic intensity peaks. A high‐symmetry reference lattice was reconstructed by inverse FFT using masked principal frequencies corresponding to the average periodic lattice. Local displacement vectors and their magnitude distributions were then obtained by measuring the deviation of experimental atomic positions from the ideal reference lattice. Specimens for EBSD, TKD, and TEM characterization were electropolished in a twin jet system with an electrolyte containing 90 vol% ethyl alcohol and 10 vol% perchloric acid at −20°C. The XRD measurements conducted on a Bruker‐AXS D8 Advance diffractometer with Cu‐Kα radiation, with a scanning speed of 1° per minute.

### 5.3 Tensile Testing

Flat dog‐bone‐shaped tensile specimens with a gauge cross‐section of 2.5 × 1 mm and a length of 10 mm were electro‐discharge machined. Tensile tests were performed with an LTM‐20KN testing machine at a constant strain rate of 1 × 10^−3^ s^−1^ at liquid nitrogen temperature (77 K). Before testing, the specimens were soaked in liquid nitrogen for approximately 20 min. Three tensile specimens were prepared for each sample to ensure repeatability.

## Funding

The authors acknowledge financial supports from the National Key R&D Program of China (Grant no. 2021YFA1200203), the Fundamental Research Funds for the Central Universities (Grant no. B250201290), the National Natural Science Foundation of China (Grant nos. 92366201, 52371068, 51971112, and 51225102), the Jiangsu Province Leading Edge Technology Basic Research Major Project (Grant no. BK20222014), and the open research fund of the Suzhou Laboratory (Grant no. SZLAB‐1108‐2024‐TS002).

## Conflicts of Interest

The authors declare no conflicts of interest.

## Supporting information




**Supporting File**: advs75679‐sup‐0001‐SuppMat.docx.

## Data Availability

The data that support the findings of this study are available from the corresponding author upon reasonable request.
