# Supplementary material for: Hierarchical Ordering Induced Ultrahigh Cryogenic Strength and Strain Hardening in a Ni2CoFeV Medium‐Entropy Alloy
Source: Adv Sci (Weinh). 2026 May 11:e75679. Online ahead of print. doi: 10.1002/advs.75679 (PMC13336006; doi:10.1002/advs.75679)
Supplement: Supplementary file 1 — Supporting File: advs75679‐sup‐0001‐SuppMat.docx. [file ADVS-9999-e75679-s001.docx]

Supporting Information

**Hierarchical Ordering Induced Ultrahigh Cryogenic Strength and Strain Hardening in a Ni_2_CoFeV Medium-Entropy Alloy**

Lei Gu, Wei Jiang, Qingzhong Mao, Xiang Chen*, and Yonghao Zhao*


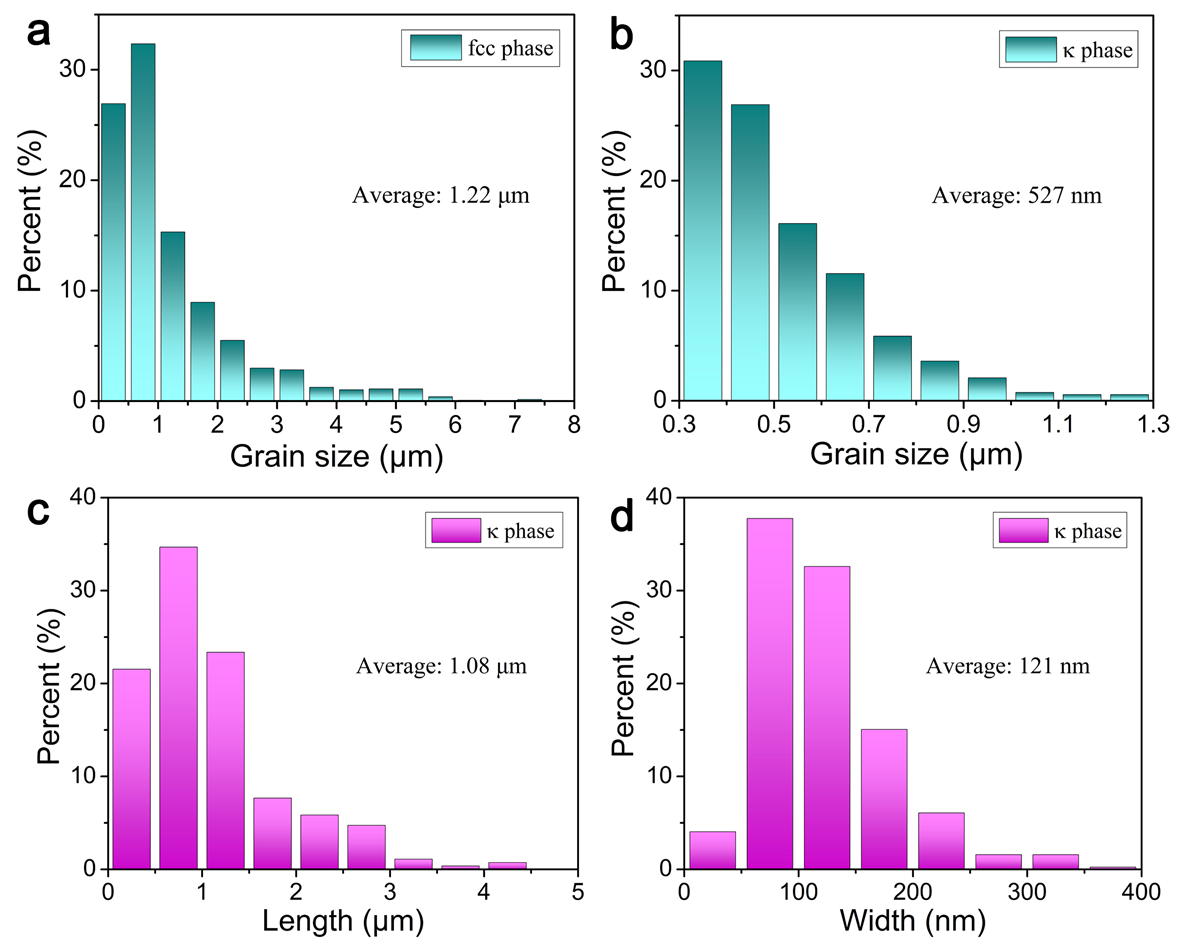


**Figure S1.** The size distributions of the tri-phase Ni_2_CoFeV MEA. a,b) The average equivalent circular diameter of the fcc and κ phases obtained from EBSD data. c,d) The length and width distributions of the κ phase measured from TEM images.


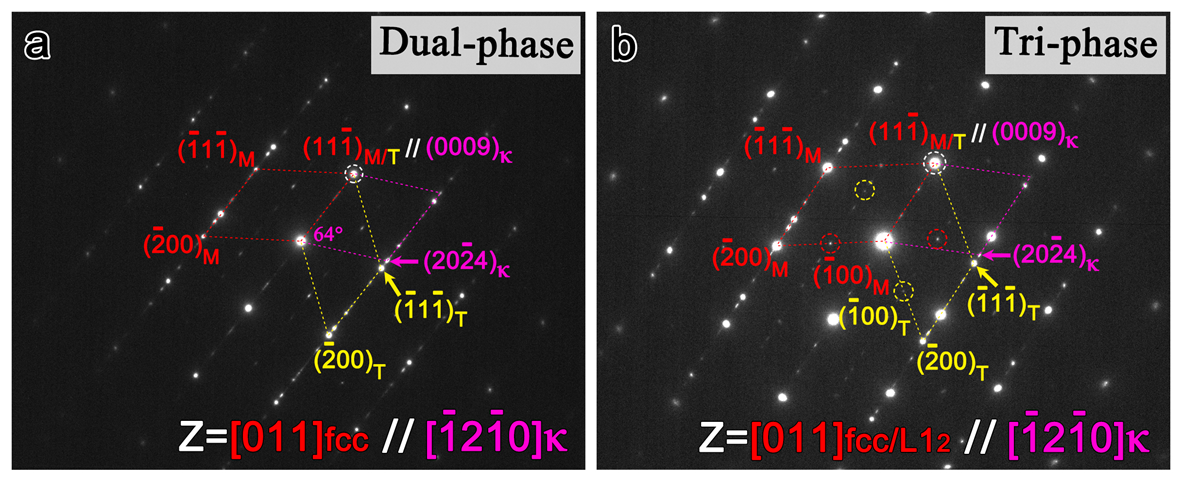


**Figure S2**. The SAED patterns of the dual-phase and tri-phase Ni_2_CoFeV MEAs. a) Dual-phase showing an orientation relationship of (0009)κ//(11$\bar{\text{1}}$)fcc and [$\bar{\text{1}}$2$\bar{\text{1}}$0]κ//[011]fcc. b) Tri-phase showing an orientation relation of (0009)κ//(11$\bar{\text{1}}$)fcc/L1_2_ and [$\bar{\text{1}}$2$\bar{\text{1}}$0]κ//[011]fcc/L1_2_.


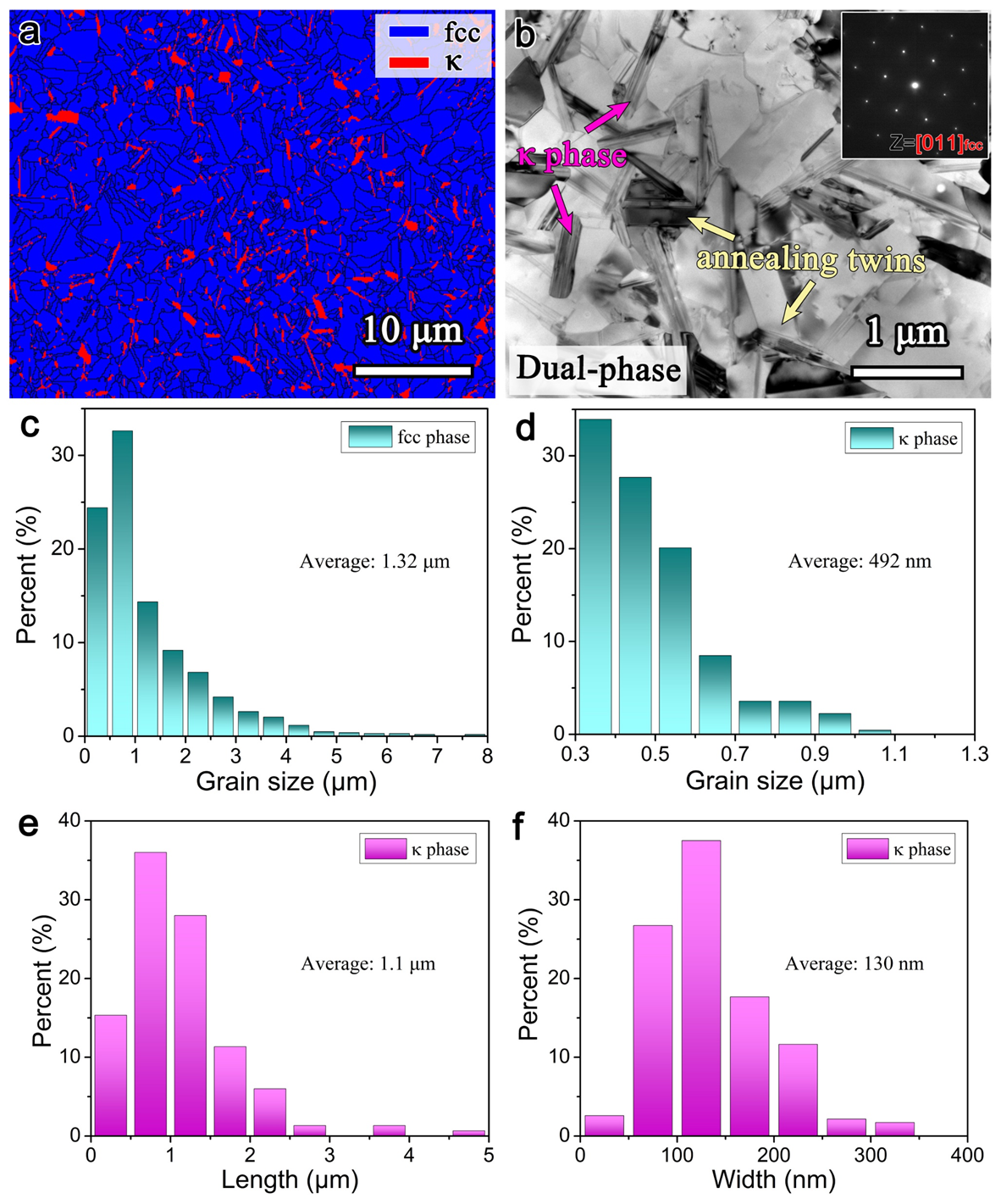


**Figure S3.** The microstructures and size distributions of the dual-phase Ni_2_CoFeV MEA. a) EBSD phase map with HAGBs marked by black lines. b) TEM image with the inserted SAED pattern of the fcc phase. c,d) The average equivalent circular diameter of the fcc and κ phases obtained from EBSD data. e,f) The length and width distributions of the κ phase measured from TEM images.


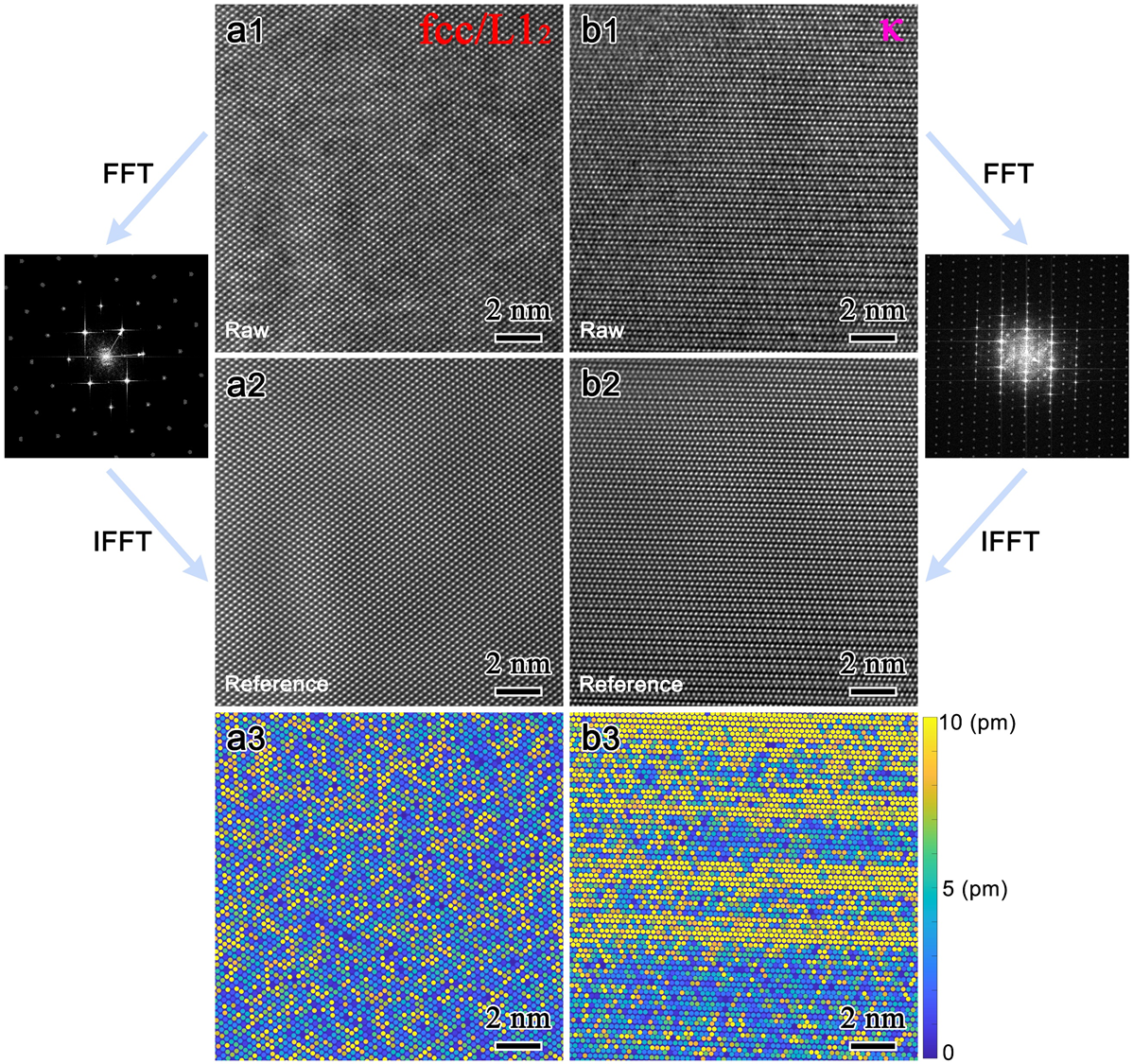


**Figure S4**. Quantitative analysis of local lattice distortion via displacement separation analysis (DSA) for the a1-a3) fcc/L1_2_ and b1-b3) κ phases in the tri-phase Ni_2_CoFeV MEA. a1-b1) Raw HRTEM images. a2-b2) Corresponding high-symmetry reference lattices reconstructed via inverse fast Fourier transform (IFFT) for DSA processing. a3-b3) Color-coded maps of atomic displacement magnitudes, revealing the distribution of local lattice distortion relative to the ideal reference lattice.


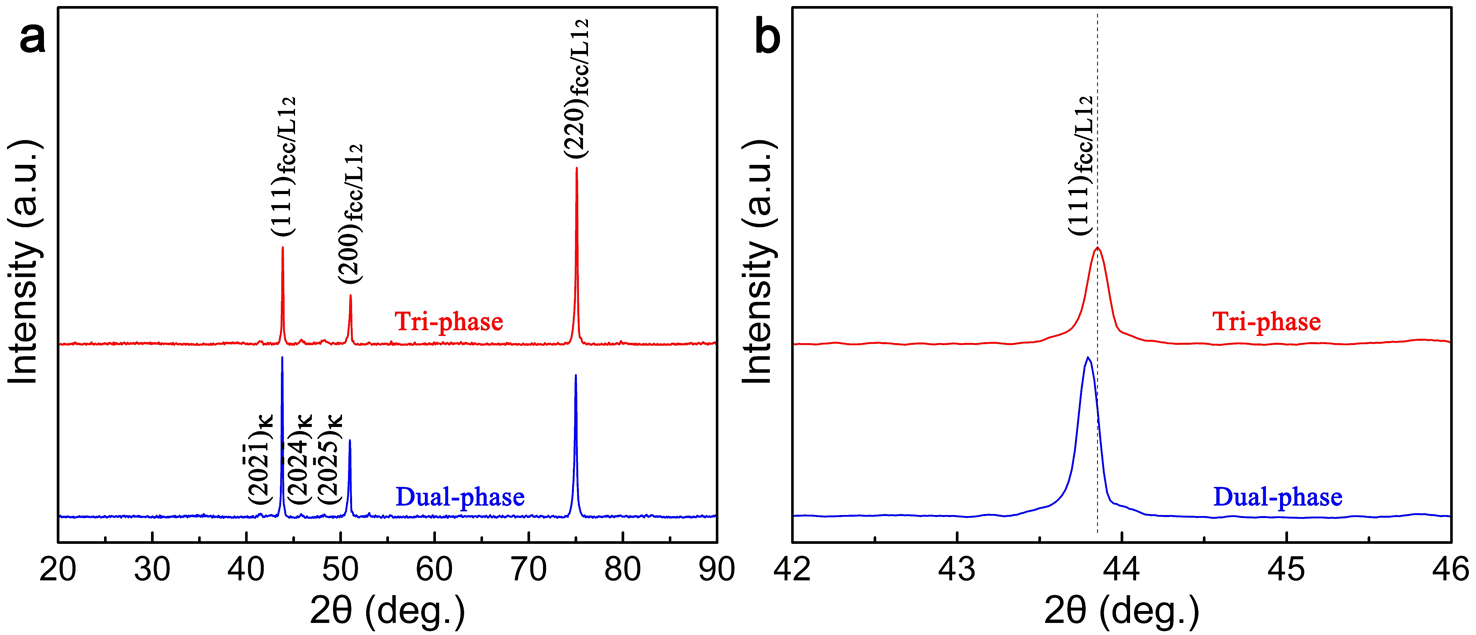


**Figure S5**. XRD patterns of the dual-phase and tri-phase MEAs.


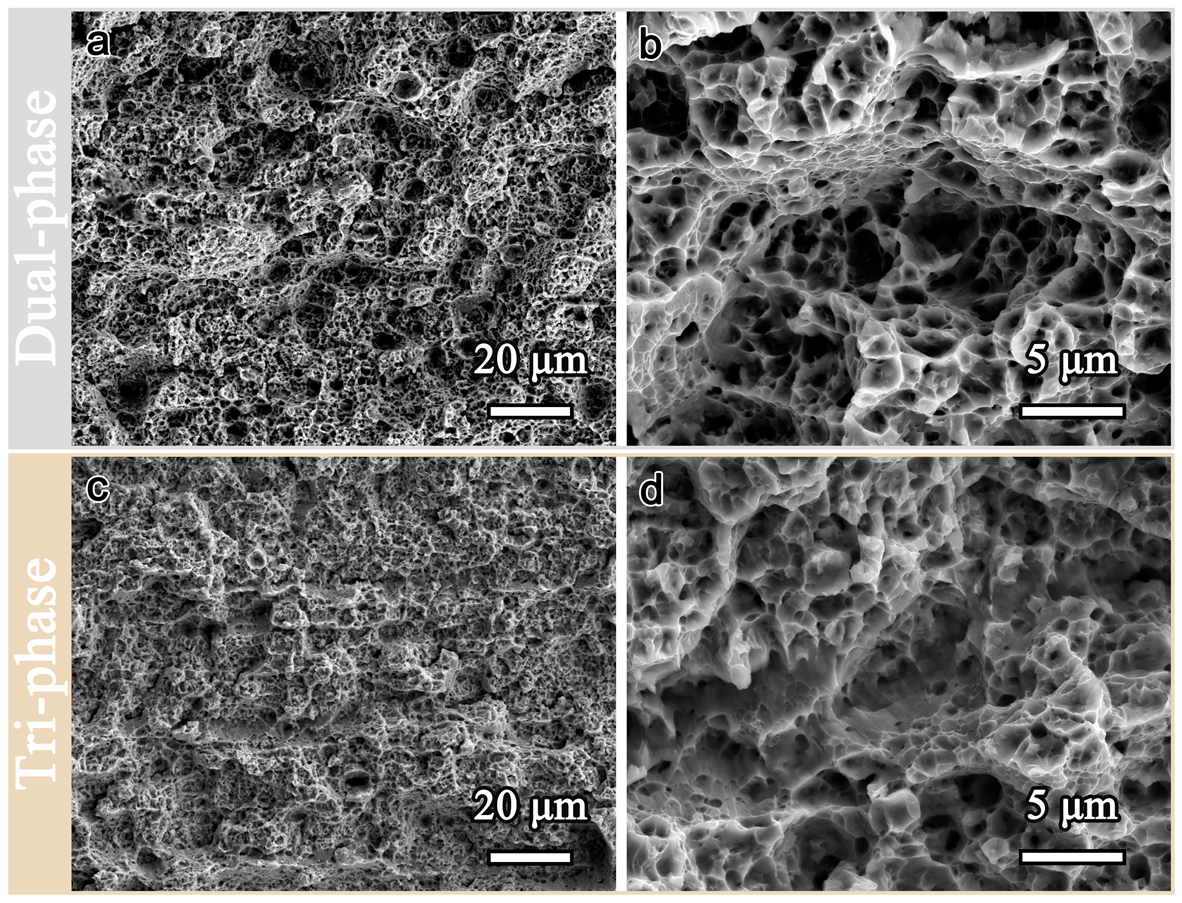


**Figure S6.** SEM images of the fracture morphology of the dual-phase and tri-phase Ni_2_CoFeV MEAs. a,b) Dual-phase. c,d) Tri-phase. The uniformly distributed, honeycomb-like dimples are observed.


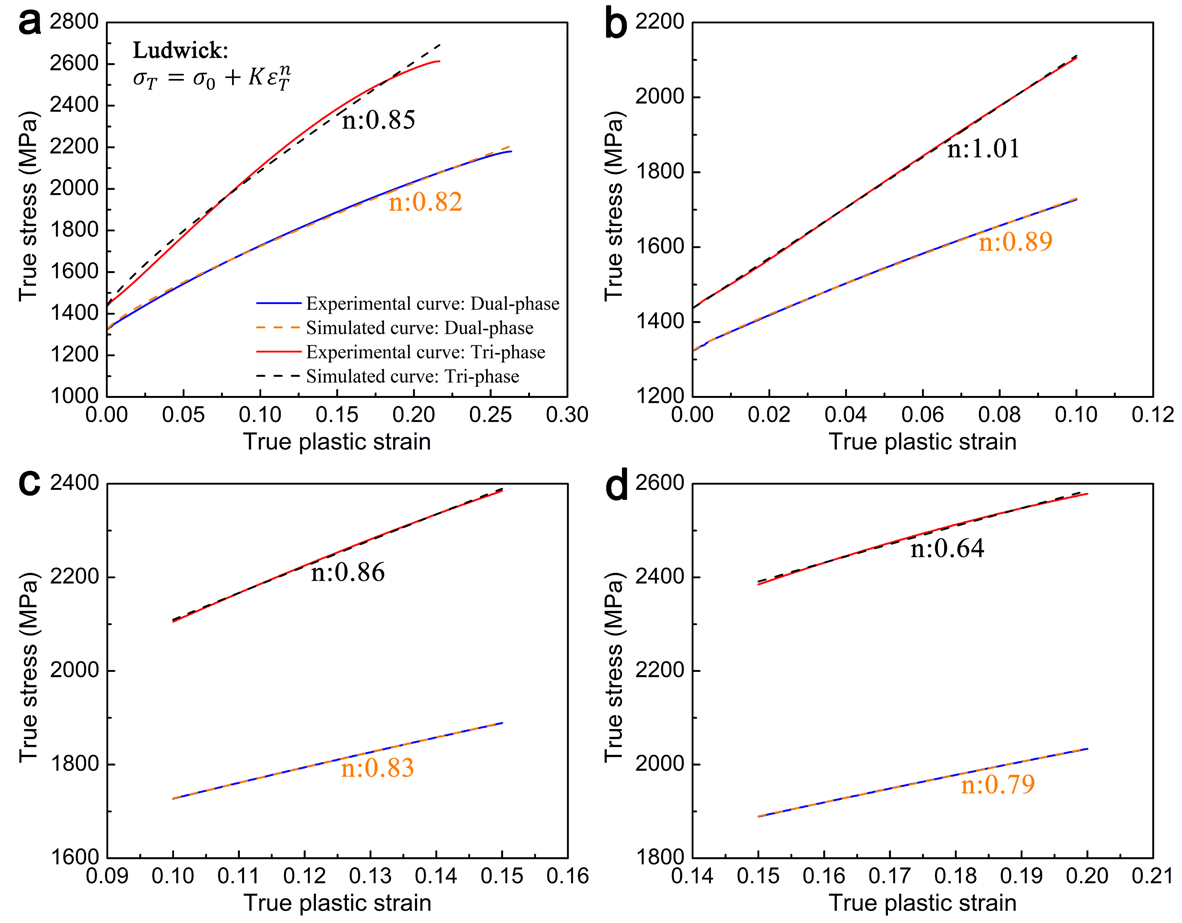


**Figure S7.** Experimental true stress versus true plastic strain curves (solid lines) and simulated curves by Ludwick equation (dashed lines). a) The whole stage. Strains of b) 0-0.1, c) 0.1-0.15 and d) 0.15-0.2.


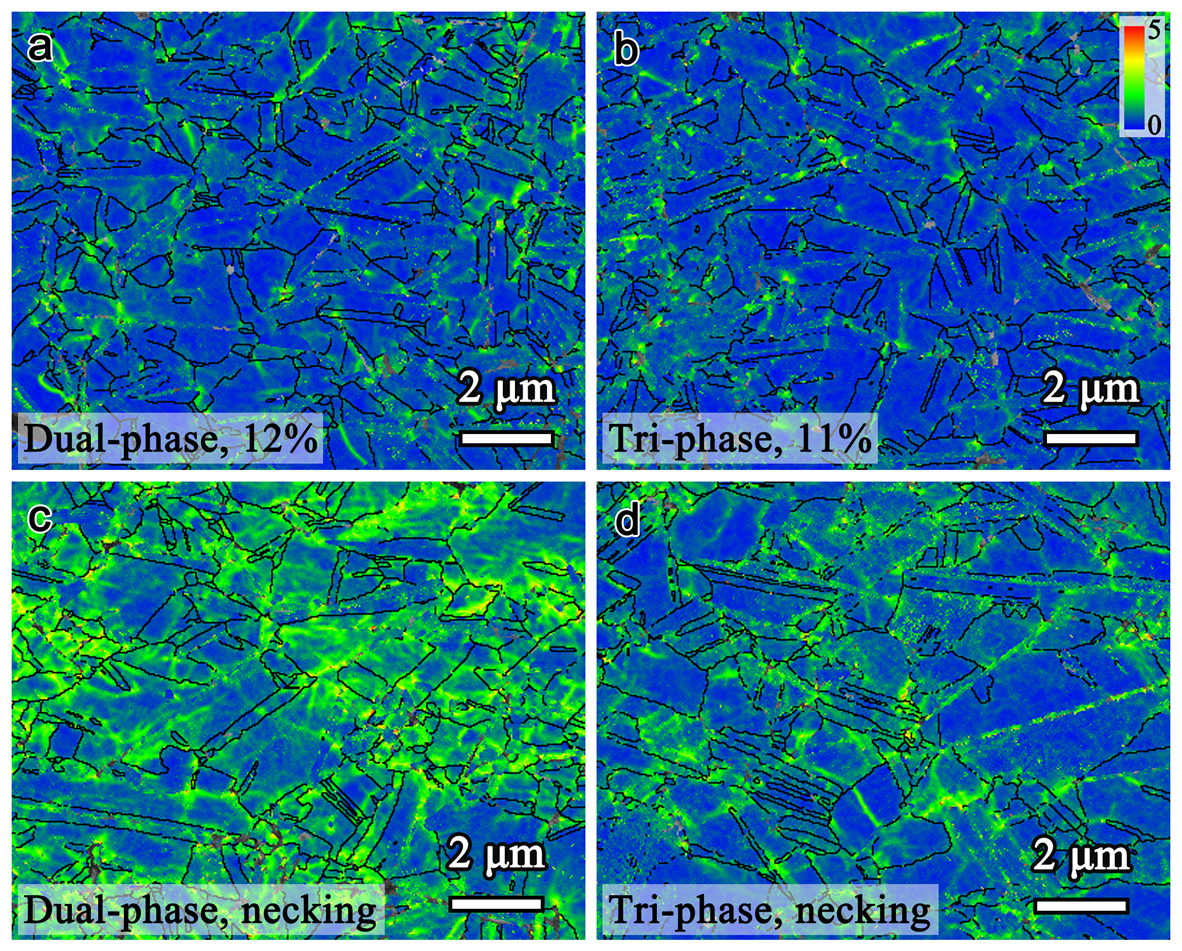


**Figure S8.** TKD local misorientation maps of the dual-phase and tri-phase MEAs tested at 77 K with different engineering strains. a,c) Dual-phase at 12% strain and necking. b,d) Tri-phase at 11% strain and necking.


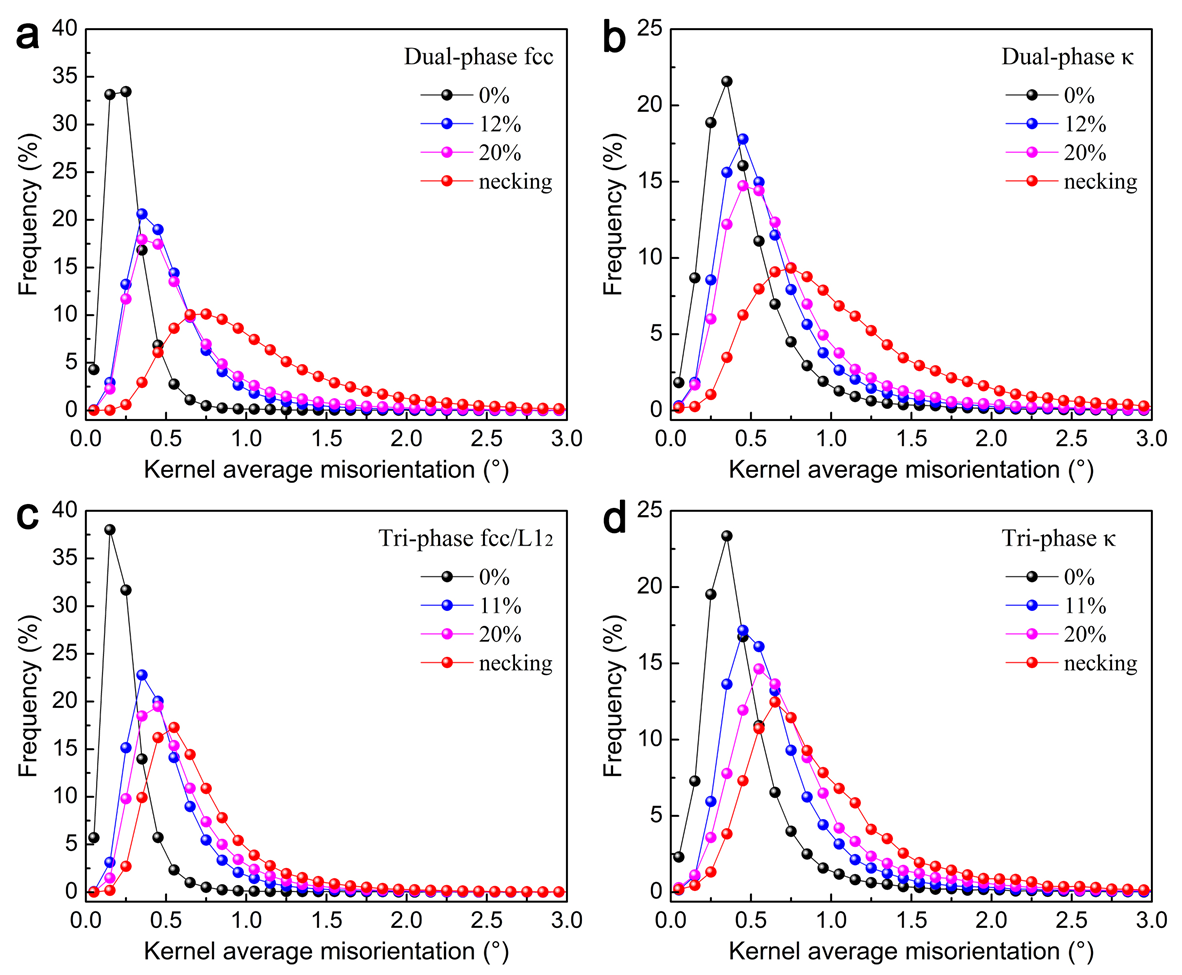


**Figure S9.** The KAM value distributions at different engineering strains. a) The fcc and b) κ phases in the dual-phase MEA. c) The fcc/L1_2_ and d) κ phases in the tri-phase MEA.


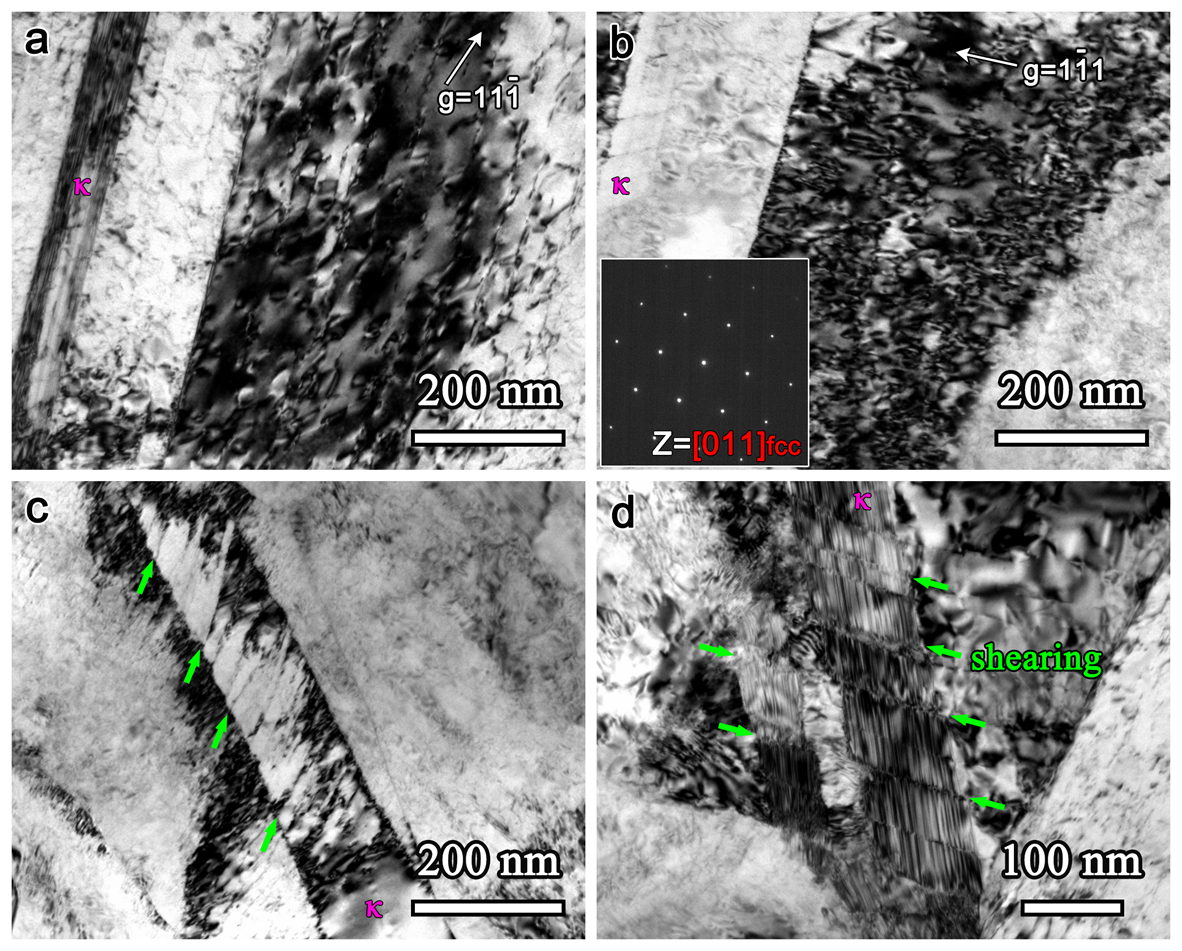


**Figure S10.** Deformation microstructures of the fractured dual-phase MEA after tensile loading at 77 K. a,b) TEM images in two-beam condition near the [011] zone axis, showing dislocation entanglements. c,d) The dislocation shearing on a single slip system dominating the κ phase indicated by green arrows.


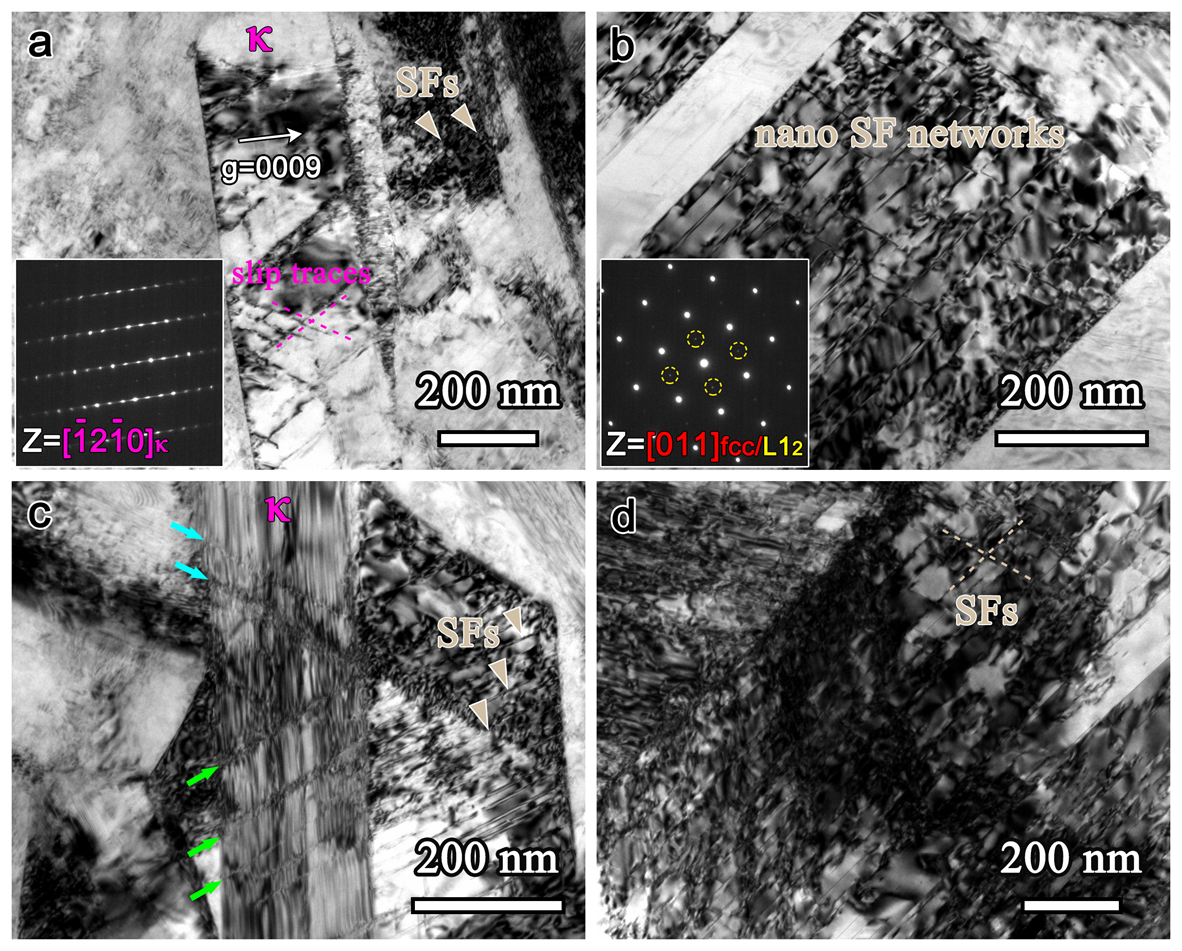


**Figure S11.** Deformation microstructures of the fractured tri-phase MEA after tensile loading at 77 K.
